# A Comparative Study of Frontal and Cerebellar Lobe Volumes Between Patients With First-Episode Schizophrenia and Healthy Controls and Its Association With Psychopathology and Neurological Soft Signs in Patients

**DOI:** 10.7759/cureus.71389

**Published:** 2024-10-13

**Authors:** Siddhant Mahapatra, Ajish Mangot, Asif Tamboli

**Affiliations:** 1 Psychiatry, Krishna Institute of Medical Sciences, Krishna Vishwa Vidyapeeth (Deemed to be University), Karad, IND; 2 Radiodiagnosis, Krishna Institute of Medical Sciences, Krishna Vishwa Vidyapeeth (Deemed to be University), Karad, IND

**Keywords:** cerebellum, cognition, frontal lobe, neurological soft signs, psychopathology, schizophrenia, volbrain, volume

## Abstract

Objectives

Our study aimed to investigate the fronto-cerebellar volumes in both patients and controls, as well as explore their relationship with symptomatology. Our primary objectives were to compare the frontal and cerebellar lobe volumetric measurements between patients with first-episode schizophrenia (FES) and healthy controls and to assess the relationship of these volumes with psychopathology, cognition, and neurological soft signs in FES patients. The secondary objective was to explore the association of fronto-cerebellar lobe volumes with socio-demographic factors among patients and controls, as well as the duration of untreated illness (DUI) among patients.

Materials and methods

This was a cross-sectional, case-control study involving 60 participants, including 30 antipsychotic-naïve FES patients and 30 healthy controls. Participants underwent MRI scanning to measure frontal and cerebellar lobe volumes using the volBrain platform. Additionally, FES patients were assessed using the Positive and Negative Syndrome Scale (PANSS), Montreal Cognitive Assessment-Basic (MoCA-B), and Brief Motor Scale (BMS). Pearson's correlation, independent sample t-tests, multivariate linear regression, and binomial logistic regression were used to analyze the relationships between brain volumes, clinical assessments, and socio-demographic factors.

Results

No significant differences in frontal volumes were found between the two groups, while cerebellar volumes were significantly smaller in FES patients (p=0.004), particularly in younger males (p=0.026). Frontal volumes were negatively correlated with age in both groups (p=0.012), which remained robust in patients even after controlling for their gender, education, and DUI (p=0.012, aR2=0.221). Cerebellar volume reduction was associated with a higher likelihood of being classified as a patient (p=0.029).

BMS was significantly correlated with frontal lobe volumes, especially in motor sequencing (MoSe), after adjusting for age, gender, and education (p=0.009). BMS MoSe scores were also significantly positively correlated with the DUI (r=0.415, p=0.023) and PANSS-General Psychopathology (GP) (r=0.494, p=0.005). MoCA-B scores were significantly lower in females than males (p=0.016), while PANSS-GP was significantly negatively correlated with age (r=-0.432, p=0.017).

Conclusion

Frontal and cerebellar volumes were differentially impacted in FES, with cerebellar atrophy being a significant distinguishing feature of the disorder. Frontal atrophy was associated with motor dysfunction but did not appear to influence psychopathology or cognition significantly in the early stages. The independent effects of frontal and cerebellar volumes, as shown by the lack of correlation between them, may suggest that these brain regions undergo separate pathological processes in schizophrenia, with frontal functional changes impacting motor function and cerebellar structural changes contributing to broader psychiatric symptoms, thereby warranting further exploration involving larger sample size.

## Introduction

Schizophrenia is a severe and chronic psychiatric disorder that affects approximately 0.3% of the global population [1]. It is characterized by a constellation of symptoms, including positive symptoms (such as hallucinations and delusions), negative symptoms (such as diminished motivation and affect), and cognitive impairments. These symptoms can profoundly disrupt an individual's ability to function in daily life, leading to significant disability and social isolation. Schizophrenia typically manifests in late adolescence or early adulthood, with a slightly earlier onset in males compared to females [2]. Its etiology is complex and multifactorial, involving a combination of genetic, environmental, and neurodevelopmental factors [3]. The neurodevelopmental hypothesis of schizophrenia posits that these genetic and environmental factors disrupt early brain development, leading to structural and functional brain abnormalities that predispose individuals to schizophrenia [3].

Advancements in neuroimaging techniques, particularly magnetic resonance imaging (MRI), have provided valuable insights into the structural and functional brain abnormalities associated with schizophrenia. One of the most consistent findings is the reduction in gray matter volume in several brain regions, including the cerebellum and frontal lobes [4,5]. These regions are critical for cognitive and emotional processing, and their dysfunction is thought to underlie many of the symptoms of schizophrenia.

The cerebellum and its connections have been specifically associated with neurological soft signs (NSS), which are subtle motor and sensory impairments frequently observed in schizophrenia, reflecting underlying neurodevelopmental abnormalities [6]. Moreover, these reductions in cerebellar volume have also been linked to more severe cognitive deficits and psychopathological symptoms, further implicating the cerebellum in the broader symptomatology of schizophrenia [4]. The concept of cerebellar cognitive affective syndrome (CCAS) has emerged as a framework for understanding the cerebellum's contributions to cognitive and emotional processing [7]. CCAS is characterized by impairments in executive function, language, visuospatial abilities, and affect regulation, which overlap significantly with the symptoms seen in psychosis and schizophrenia [8].

Similarly, reductions in frontal lobe volume have been linked to increased NSS, cognitive impairments, and more severe psychopathological symptoms in schizophrenia [9-11]. The frontal lobes, particularly the prefrontal cortex, are involved in executive functions, such as planning, decision-making, and impulse control, all of which are often impaired in individuals with schizophrenia.

There is a significant lack of studies from India examining the association between brain volumes and NSS, psychopathology, and cognition. One study from India investigating brain volumetric abnormalities in recent-onset schizophrenia did not report any notable abnormalities in whole-brain or regional brain morphometry [12]. Given the limited and sometimes contradictory literature, we aimed to investigate the fronto-cerebellar volumes in both patients and controls, as well as explore their relationship with symptomatology.

Our primary objectives were to compare the frontal and cerebellar lobe volumetric measurements between patients with first-episode schizophrenia (FES) and healthy controls and to assess the relationship of these volumes with psychopathology, cognition, and NSS in FES patients. The secondary objective was to explore the association of fronto-cerebellar lobe volumes with socio-demographic factors among patients and controls, as well as the duration of untreated illness (DUI) among patients.

We hypothesized that fronto-cerebellar volumes in FES patients would be reduced compared to healthy controls and that these volumes would be significantly associated with the severity of psychopathology, cognitive impairment, and NSS.

## Materials and methods

This was a case-control study that was observational, analytical, and cross-sectional. A total of 60 participants were included, comprising 30 patients with antipsychotic naïve FES and 30 healthy controls. According to Cahn et al., the mean cerebellar volumes in FES patients was 144.9 (±11.9) cm^3^, and that of healthy controls was 149.7 (±11.7) cm [13]. The sample size was estimated using the below-mentioned formula:

$n=\frac{\left(S D_1^2+S D_2^2\right)\left(Z_{1-\frac{a}{2}}+Z_{1-b}\right)^2}{\left(M_1-M_2\right)^2}=\frac{\left(11.9^2+11.7^2\right) \times 7.84}{(144.9-149.7)^2}=95$

Thus, the total sample size based on generalizability and adequacy of power was 190 (95 patients + 95 controls). However, based on the number of FES patients expected to visit our outpatient department for the study duration of 18 months and the financial feasibility, the final total recruited sample size was 60 (30 patients + 30 controls).

Participants were recruited from our psychiatry outpatient department, with patients meeting the inclusion criteria of being antipsychotic-naïve adults aged 18-45 years with a diagnosis of schizophrenia, as per the World Health Organization's International Classification of Diseases 11th Revision. Exclusion criteria included other psychiatric comorbidities, intellectual disability, and any medical or surgical morbidity. Age and gender-matched physically healthy controls without present, past, or family history of mental health ailments were recruited. The institutional ethical committee had approved the protocol, and written informed consent was taken from all participants in a language and manner that they best understood.

For both FES patients and healthy controls, brain MRI scans were performed using a 1.5 Tesla MAGNETOM Avanto MRI Scanner (Siemens Healthineers, Erlangen, Germany) available in our institute. The T1-weighted three-dimensional magnetization prepared rapid acquisition gradient echo (MP-RAGE) sequence was chosen for its high resolution and contrast, which is essential for volumetric brain analysis. MRI data were analyzed using the volBrain platform, an online automated system that facilitates accurate and reproducible brain segmentation. The MP-RAGE MRI sequences were converted to the Neuroimaging Informatics Technology Initiative (NIfTI) format and processed through the vol2Brain 2.0 pipeline, which segmented the brain into 135 structures, offering detailed volumetric measurements and cortical thickness analysis. The volBrain platform was chosen because it was online, free, did not require any technical expertise and results generated are comparable with other offline publicly available segmentation tools [14].

In addition to the brain volumetric measurement, FES patients underwent comprehensive assessments.

Positive and Negative Syndrome Scale (PANSS) 

The PANSS is a standardized clinical interview used for rating positive symptoms, negative symptoms, and general psychopathology (PANSS-GP) for schizophrenia patients within the last week. It has seven items to rate positive symptoms, seven for negative symptoms, and 16 for general psychopathology symptoms, with each item rated along a seven-point scale (1=absent; 7=extreme). The total score ranges from 30-210 [15].

Montreal Cognitive Assessment-Basic (MoCA-B)

MoCA is a self-administered screening tool for evaluating cognitive domains like attention, concentration, short-term memory, working memory, visuospatial abilities, orientation, executive functions, and language. It has a total of 12 questions with a total score of 30. A score less than 26 is considered abnormal. It has been specifically used for screening cognitive impairment in schizophrenia and has been found to correlate well with other established instruments for detecting cognitive impairment in schizophrenia [16]. An official Marathi-translated version 7.1 with good reliability and validity is freely available from their website. MoCA-B has adapted questions that facilitate the detection of mild cognitive impairment in subjects who are illiterate or who possess less than five years of education [17]. Before administering to patients, the first author of our paper underwent official training and certification. 

Brief Motor Scale (BMS)

BMS is used for the assessment of motor soft signs in schizophrenia and other psychiatric disorders. It has high sensitivity (84.1%) and specificity (87.9%). It has a total of 10 items involving motor coordination and motor sequencing, with a total score ranging from 0-20 [18].

Free and open-source statistical software, Jamovi version 2.3.28, was used for analysis. Pearson's correlation, independent samples t-test, multivariate linear regression, and binomial logistic regression were employed to test the association between variables. A p-value of less than or equal to 0.05 was considered significant.

## Results

Socio-demographic factors

The mean age of patients and controls were 30.7 years (±6.67) and 29.8 years (±5.54) respectively. There were 14 (47%) and 13 (43%) males among patients and controls, respectively, while there were 16 (53%) and 17 (57%) females, respectively. Fourteen (53%) patients were educated beyond grade X, while in the control group 29 (97%) had an educational level beyond grade X. While both groups were comparable in terms of age (X^2^(1, N=60) = 1.11,p=0.292) and gender (X^2^(1, N=60) = 0.0673, p=0.795) distribution, education was skewed. The median DUI among patients was 10 weeks, with a wide variation ranging from one to 104 weeks.

Fronto-cerebellar volumes with socio-demographic factors

Mean frontal lobe volumes were 164.73 (±22.86) cm^3^ in patients and 172.26 (±17.56) cm^3^ in controls. Mean cerebellar volumes were 111.14 (±12.20) cm^3^ in patients and 124.72 (±15.15) cm^3^ in controls. To account for gender-based differences, the percentage of frontal and cerebellar volumes relative to total brain volume (TBV) was used instead of absolute values for their comparison [19]. Accordingly, the mean frontal volume percentage with respect to TBV was 13.40 (±1.02) in patients and 13.20 (±0.86) in controls, while the mean cerebellar volume percentage with respect to TBV was 8.93 (±0.79) in patients and 9.58 (±0.91) in controls.

In patients, frontal volumes were significantly negatively correlated with age (r=-0.539, p=0.002; Table 1), a relationship that remained robust even after controlling for gender, education, and the DUI in the multivariate linear regression model (p=0.012, aR^2^=0.221; Table 2). In controls, frontal volumes were negatively correlated with age, but the association became significant only when adjusted for education and gender. In contrast, cerebellar volumes showed no significant correlations with age, gender, or education in either group. Also, no significant correlation was found between frontal and cerebellar volumes in either group. 

**Table 1:** Correlation matrix in patients Pat_DUI - Patients' duration of untreated illness; PANSS_GP - Positive and negative syndrome scale (general psychopathology score); BMS_MoCo - Brief motor scale (motor coordination); BMS_MoSe - Brief motor scale (motor sequencing) Pearson's correlation; *p<0.05

Variables	Correlation	Age	Pat_DUI	PANSS_GP	BMS_MoCo	BMS_MoSe
PANSS_GP	Pearson's r	-0.432	—	—	—	—
	p-value	0.017*	—	—	—	—
BMS_MoSe	Pearson's r	—	0.415	0.494	0.425	—
	p-value	—	0.023*	0.005*	0.019*	—
Frontal volume percentage	Pearson's r	-0.569	—	—	0.423	0.480
	p-value	0.001*	—	—	0.020*	0.007*

**Table 2:** Frontal volume in patients with respect to age, gender, education, and duration of untreated illness M - male; F - female; Edu_Cat - educational category (above grade X and up to grade X); DUI - patients' duration of untreated illness

Model fit measures		Predictor	Estimate	SE	t	p-value
R	0.573	Intercept	15.6389	0.99777	15.6739	
		Age	-0.0785	0.02885	-2.7206	0.012*
R²	0.328	Gender: M-F	0.0174	0.31929	0.0546	0.957
		Edu_Cat: Above X – Upto X	0.1446	0.36745	0.3934	0.697
Adjusted R²	0.221	DUI	5.47e-4	0.00545	0.1004	0.921
		Model coefficients (frontal volume percentage) - multivariate linear regression; *p<0.05				

When comparing fronto-cerebellar volumes between patients and controls, cerebellar volumes were significantly smaller in patients compared to controls (p=0.004, Cohen's d =-0.770, 95% CI (-1.308, -0.221), indicating a moderate effect size), particularly among males (p=0.026, Cohen's d = -0.913, 95% CI (-1.73, -0.068), indicating a large effect size) and those aged 18-30 years (p=0.006, Cohen's d = -0.984, 95% CI (-1.72, -0.227), indicating a large effect size; Table 3). The binomial logistic regression revealed that smaller cerebellar volumes were significantly associated with a higher likelihood of being a patient (p=0.029; Table 4). However, no significant differences were found with respect to frontal volumes.

**Table 3:** Fronto-cerebellar volumes in patients vs controls Independent samples t-test; *p <0.05

Variables	Patients vs controls	Number (N)	Mean (%)	Standard deviation	Statistic	p-value
Frontal volume	Patients	30	13.3	0.98	0.506	0.615
	Controls	30	13.2	0.865		
Cerebellar volume	Patients	30	8.92	0.804	-2.983	0.004*
	Controls	30	9.58	0.91		
Cerebellar volume in 18-30 year age group	Patients	16	8.81	0.965	-2.93	0.006*
	Controls	20	9.71	0.875		
Cerebellar volume in males	Patients	14	8.69	0.924	-2.37	0.026*
	Controls	13	9.6	1.069		

**Table 4:** Fronto-cerebellar volumes in patients vs controls with respect to age, gender, and educational status Age_Cat - age category (18-30 years and 31-45 years); M - male; F - female; Edu_Cat - education category (above grade X and up to grade X) Model coefficients - binomial logistic regression; estimates represent the log odds of "group = patient" vs. "group = control"; *p<0.05

Model fit measures		Predictor	Estimate	SE	Z	p-value
Deviance	50.3	Intercept	8.743	7.95	1.1	0.271
		Frontal volume percentage	0.617	0.421	1.467	0.142
AIC	62.3	Cerebellar volume percentage	-1.418	0.649	-2.186	0.029*
		Age_Cat: 31-45 – 18-30	-1.045	0.951	-1.099	0.272
R²_McF_	0.396	Gender: M-F	0.342	0.737	0.464	0.643
		Edu_Cat: above X - up to X	-4.656	1.362	-3.418	

Psychopathology with socio-demographic factors

PANSS-GP was significantly negatively correlated with age (r =-0.432, p=0.017; Table 1), which remained robust after adjusting for the influence of gender, education, and DUI (p=0.024, aR^2^=0.175). Male MoCA scores (27.9 ±1.75) were significantly higher (p=0.016, Cohen's d =-0.94, 95% CI (-1.72, -0.136), indicating large effect size) than female scores (25.4 ± 3.12). This difference remained significant even after adjusting for the influence of age, education, and the DUI (p=0.023, aR^2^=0.087). BMS MoSe scores were significantly positively correlated with DUI (r=0.415, p=0.023; Table 1), which remained robust after adjusting for the influence of age, gender, and education (p=0.006, aR^2^=0.239). BMS MoSe scores were also significantly positively correlated with PANSS-GP (r=0.494, p=0.005).

Fronto-cerebellar volumes with psychopathology

In patients, frontal volumes were positively correlated with BMS MoCo (r = 0.423, p = 0.020) and MoSe scores (r=0.480, p=0.007; Table 1), suggesting that frontal lobe volumes may contribute to NSS. This relationship remained significant only for motor sequencing after adjusting for age, gender, and education, indicating that the frontal lobe is an independent factor associated with NSS (p=0.009; Table 5). Frontal volumes did not show any significant association with PANSS and MoCA scores. Cerebellar volumes were not associated with any of the psychopathology scores.

**Table 5:** Brief motor scale (motor sequencing) vs. frontal volumes with respect to age, gender, educational status, and duration of untreated illness BMS_MoSe - brief motor scale (motor sequencing); DUI - patients' duration of untreated illness; M - male; F - female; Edu_Cat - education category (above grade X and up to grade X) Model coefficients (BMS_MoSe) - multivariate linear regression; *p<0.05

Model fit measures		Predictor	Estimate	SE	t	p-value
R	0.712	Intercept	-2.1342	6.7617	-0.316	0.755
		Frontal volume percentage	1.1612	0.4119	2.819	0.009*
R²	0.507	Age	-0.0584	0.0676	-0.863	0.397
		DUI	0.0371	0.0112	3.308	0.003*
Adjusted R²	0.405	Gender: M-F	-0.8812	0.6576	-1.34	0.193
		Edu_Cat: above X – up to X	-0.493	0.7591	-0.649	0.522

## Discussion

Our study compared frontal and cerebellar lobe volumes between FES patients and healthy controls, exploring their associations with psychopathology, cognition, and NSS. Our findings add to the growing evidence of fronto-cerebellar involvement in schizophrenia and the complex relationships between brain structure and clinical outcomes.

The patient and control groups were comparable in terms of age and gender distribution, ensuring that these factors did not bias the results. However, education was skewed, with most controls having a higher educational level. This difference could impact cognitive measures, as those with higher education tend to perform better on assessments like the MoCA [20]. This factor was controlled for in regression analyses, as well as by the use of MoCA-B [17].

Frontal volume and socio-demographic factors

In this study, no significant differences in frontal lobe volumes were observed between FES patients and healthy controls. Current research literature suggests that the structural changes in these regions may occur independently in schizophrenia, with initial findings involving smaller brain structures like the insula and striatum in the early stages of schizophrenia may gradually evolve to further, widespread anatomical changes as the illness progresses [21]. Differences in image analysis techniques and the lower median DUI noted in our study could also have contributed to this finding.

We found a significant negative correlation between frontal volumes and age in patients, which persisted even after adjusting for gender, education, and the DUI, indicating that age-related volumetric decline may be more pronounced and occur earlier in the disorder, suggesting a larger brain age gap in patients with schizophrenia [22]. In healthy controls, this association only became significant when controlling for gender and education, emphasizing the need for considering socio-demographic factors in brain volume studies.

The lack of significant differences in frontal volumes between patients and controls contrasts with studies that have reported frontal lobe atrophy in schizophrenia [23]. However, the negative correlation between frontal volumes and age suggests that progressive changes may develop over time, particularly in more advanced stages of the disorder, consistent with the neurodevelopmental and progressive nature of schizophrenia [3,21,22].

Cerebellar volume and socio-demographic factors

Cerebellar volumes were significantly reduced in FES patients compared to controls, especially among males and those aged 18-30 years. This finding supports existing research highlighting the cerebellum’s role in schizophrenia, not only in motor functions but also in cognitive and emotional regulation [4,7,8]. Lower cerebellar volumes among younger males observed in our study suggest a complex interplay between age and gender in the neuro-maturational substrates of psychosis [24]. The logistic regression also revealed that smaller cerebellar volumes were significantly associated with a higher likelihood of being a patient after controlling for various socio-demographic factors. These findings reinforce the cerebellum as a critical site of structural alteration in schizophrenia, consistent with previous studies [4].

Frontal and cerebellar volume relationships

On comparing frontal and cerebellar volumes, no significant correlations were found between the two regions. This suggests that volume reductions in the cerebellum are likely independent of changes in the frontal lobe in the early stages of schizophrenia. These findings are consistent with the notion that schizophrenia affects multiple brain regions through distinct pathological processes rather than uniformly impacting the brain [21].

Associations with psychopathology, neurological soft signs, and cognition

The relationship between brain volumes and clinical measures of psychopathology, cognition, and NSS revealed important insights. Frontal volumes in FES patients were significantly positively correlated with NSS, both the sequencing and coordination components of the BMS. This supports the idea that frontal lobe integrity is associated with motor coordination and neurological function in schizophrenia [6,9]. This relationship persisted even after controlling for socio-demographic factors and the DUI, highlighting a potential role of frontal structures in motor dysfunction in schizophrenia. These findings align with previous studies that have suggested fronto-cerebellar network involvement in motor deficits in schizophrenia [25]. This also suggests that although anatomical alterations like smaller cerebellar volumes may be observed earlier, functional alterations may be more apparent in frontal lobes especially in FES.

There were no significant associations between frontal volumes and PANSS scores, suggesting that frontal atrophy may not directly contribute to the severity of psychotic symptoms in early-stage schizophrenia. The relationship with MoCA was also not significant, indicating that cognitive impairments may not be strongly linked to frontal lobe changes in this patient population, contrasting other findings [11].

The finding that cerebellar volumes were not significantly associated with psychopathology measures contradicts studies that have implicated the cerebellum in cognitive functions, NSS, and transdiagnostic behavioral dimensions of psychopathology, especially general psychopathology and executive dysfunction [4,26]. These discrepancies may be due to differences in study design, sample size, and disease stage.

Psychopathology and socio-demographic factors

We found PANSS-GP to be significantly negatively correlated with age, which remained robust after adjusting for the influence of gender, education, and DUI. This probably indicates greater severity of symptomatology in the younger age group, suggesting a poorer prognosis. Also, differential age effects on psychopathology may be explained by differential brain maturation and neurodegenerative processes [27].

Our finding of females having higher cognitive impairment than males contrasts with Zhao et al. findings of higher cognitive impairment among males and Chen et al., who found no significant gender-based difference in cognitive function [28,29]. This suggests that gender differences in cognitive impairment among schizophrenia patients have yielded mixed results and need further exploration.

Our finding of BMS MoSe scores being significantly positively correlated with the DUI and PANSS-GP is like the findings by Petrescu et al., where NSS was significantly associated with the course and psychopathology of schizophrenia, thus emphasizing their potential as reliable assessment tools in both research and clinical settings [30].

Our findings reinforce the need for future research to explore the distinct roles of these brain regions in schizophrenia, with a particular focus on how these structural changes relate to the progression of clinical symptoms. Understanding these independent pathophysiological processes affecting different brain regions could provide insights into targeted treatments for specific symptoms.

Limitations and strengths

Our study has its inherent limitations. Education level among patients and controls was skewed but was controlled by applying regression models wherever applicable, along with MoCA-B for cognitive assessment. Our relatively small sample size of 30, instead of the calculated number of 95, may limit the generalizability of the findings, and the cross-sectional nature of the study prevents conclusions about causality. Further MRI-based measurement precision and variability between scanning protocols among different studies may also be limiting factors.

Despite these limitations, our use of high-resolution MRI and automated volumetric analysis provides robust and reproducible measurements. Furthermore, controlling for socio-demographic variables such as age and gender strengthens the reliability of our findings. Our findings add to the growing body of evidence suggesting both structural (cerebellar) and functional (frontal) abnormalities in the early stages of schizophrenia, thereby possibly acting as biomarkers.

## Conclusions

Our results indicated that frontal and cerebellar volumes were impacted differently in schizophrenia. Frontal volume reductions were linked to age-related decline, particularly in patients, and were associated with NSS. However, these changes did not appear to influence psychopathology or cognition significantly. In contrast, cerebellar volume reductions were a distinguishing feature, being smaller in patients than controls and contributing to a higher likelihood of patient classification.

The independent effects of frontal and cerebellar volumes, as shown by the lack of correlation between them, may suggest that these brain regions undergo separate pathological processes in schizophrenia, with frontal functional changes impacting motor function and cerebellar structural changes contributing to broader psychiatric symptoms, thereby warranting further exploration involving larger sample size.
